# Design of a VLP-nanovehicle for CYP450 enzymatic activity delivery

**DOI:** 10.1186/s12951-015-0127-z

**Published:** 2015-10-09

**Authors:** Lorena Sánchez-Sánchez, Alejandro Tapia-Moreno, Karla Juarez-Moreno, Dustin P. Patterson, Ruben D. Cadena-Nava, Trevor Douglas, Rafael Vazquez-Duhalt

**Affiliations:** Instituto de Biotecnología, Universidad Nacional Autónoma de México, 62250 Cuernavaca, Morelos Mexico; Centro de Nanociencias y Nanotecnología, Universidad Nacional Autónoma de México, Km 107 carretera Tijuana-Ensenada, 22860 Ensenada, Baja California Mexico; Cátedras CONACyT affiliated to CNyN-UNAM, Ensenada, Mexico; Department of Chemistry and Biochemistry, University of Texas at Tyler, Tyler, 75799 TX USA; Department of Chemistry, Indiana University, Bloomington, IN 47405 USA

**Keywords:** Enzymatic delivery, Cytochrome P450, Virus-like particles, Nanobioreactor, Enzyme produg therapy

## Abstract

**Background:**

The intracellular delivery of enzymes for therapeutic use has a promising future for the treatment of several diseases such as genetic disorders and cancer. Virus-like particles offer an interesting platform for enzymatic delivery to targeted cells because of their great cargo capacity and the enhancement of the biocatalyst stability towards several factors important in the practical application of these nanoparticles.

**Results:**

We have designed a nano-bioreactor based on the encapsulation of a cytochrome P450 (CYP) inside the capsid derived from the bacteriophage P22. An enhanced peroxigenase, CYPBM3, was selected as a model enzyme because of its potential in enzyme prodrug therapy. A total of 109 enzymes per capsid were encapsulated with a 70 % retention of activity for cytochromes with the correct incorporation of the heme cofactor. Upon encapsulation, the stability of the enzyme towards protease degradation and acidic pH was increased. Cytochrome P450 activity was delivered into Human cervix carcinoma cells via transfecting P22-CYP nanoparticles with lipofectamine.

**Conclusion:**

This work provides a clear demonstration of the potential of biocatalytic virus-like particles as medical relevant enzymatic delivery vehicles for clinical applications.

## Background

The therapeutic use of enzymes is a fast growing field that is the focus of extensive research from several groups and pharmaceutical companies worldwide. The interest in this class of biopharmaceuticals has dramatically increased and it is changing the way several diseases will be treated, since their mode of action involves highly specific and efficient catalysts. Therapeutic enzymes have been proposed to treat several illnesses including genetic diseases, infectious diseases and cancer [1–3]. A great proportion of the enzymes that are already in use work extracellularly; nevertheless to efficiently treat the above mentioned diseases, in particular genetic disorders and cancer, enzymes have to be internalized within the cell to reach their therapeutic target. Two significant therapeutic approaches based on enzymes have been proposed. In Enzyme Replacement Therapy (ERT) [4–6] an enzyme is exogenously provided to replace a missing enzyme, while in Enzyme Prodrug Therapy (EPT) [7–9] an enzyme capable of activating a pro-drug into its active metabolites is delivered. In both of these therapies there is a need for suitable vehicles to deliver these therapeutic enzymes intracellularly to targeted cells, while avoiding rapid inactivation and elimination from the body [10].

Virus-like particles (VLPs) offer an interesting platform as potential therapeutic agents for such intracellular delivery of enzymatic activity for several reasons including their ability to load and transport significant quantities of enzymes, their intrinsic capacity to protect their cargo and the ease with which they can be chemically and genetically modified [11]. Until now the encapsulation of enzymes inside VLPs has been useful to study biocatalysis in confined environments. Sequestration of enzymes within these vehicles can lead to high internal concentrations of cargo, in the mM range, difficult to reach with enzymes free in solution. Additionally, with the confinement of enzymes inside these viral nanoparticles, new features are generated in the resulting nanoreactor, such as improvements in stability towards temperature or protease degradation [12–18].

One of the most well studied VLPs is the one derived from the bacteriophage P22. It is a 58 nm icosahedral capsid composed of 420 coat proteins (CP) that assembles with the aid of 60–300 scaffold proteins (SP) [19]. It has been used to encapsulate several enzymes with high cargo density [15–18]. The strategy used to incorporate the enzymes into the interior of the P22 capsid requires genetically fusing the desired enzyme to the N-terminus of a truncated form of the scaffold protein, which is still capable of interacting non-covalently with the interior of CP and directing capsid assembly [20].

The aim of the work presented here was to encapsulate a cytochrome P450 (CYP), which belongs to a family of medically and industrially important enzymes, to create a nanovehicle with high catalytic activity. The variant “21B3” of CYPBM3 from *Bacillus megaterium*, with improved peroxigenase activity [21], was used as a model of this family of enzymes since it is stable and soluble in aqueous media, and it can be produced in large quantities, in contrast to human CYPs. Moreover, several mutants of this CYPBM3 have been generated which facilitate the enzymatic transformation of non-natural substrates such as pesticides [22], polycyclic aromatic hydrocarbons [23], and drugs [24], suggesting potential environmental and pharmaceutical applications. Interestingly, in a previous report we found that CYPBM3 “21B3” was able to transform the potential prodrug resveratrol as well as the anti-carcinogenic prodrug tamoxifen, one of the most commonly used drugs to treat breast cancer, into their clinically active metabolites [25].

## Results and discussion

The P22 coat protein (CP) and the CYPBM3-scaffold fusion protein (CYP-SP) were heterologously expressed in *E. coli*. Two different strategies for the in vivo assembly of the VLPs with encapsulated enzyme were used; simultaneous expression of the coat protein and CYP-SP, and differential expression of the enzyme and CP. In the latter, a two-vector approach exploited the use of different inducers to drive first the expression of CYP-SP, allowing maturation of the enzyme, and then initiation of encapsulation by inducing expression of the CP. Correctly sized capsids with cargo were produced using both approaches; however, the proportion of active cytochrome was much higher when the genes were differentially expressed (Table 1). We were able to quantify the concentration of active enzyme inside the capsids estimating the amount of CYP able to form the carbon monoxide-heme Fe^II^ complex, which produces an absorbance spectrum with a maximum wavelength at 450 nm [26]. On the other hand, the total protein was estimated from the absorbance at 280 nm and multiangle light scattering data (see below). By these means we were able to distinguish between catalytically active CYP and non-active enzyme. The maximum theoretical number of encapsulated CYPs (3.3 nm hydrodynamic radius) per P22 particle, based on volume, is around 180 enzymes. Co-expression showed the highest CYP loading, near this maximum number, but with a lower percentage of active enzyme. The fast interaction of the CYP polypeptide chain with the CP could be affecting the integrity of the enzyme as showed by the large proportion of inactive enzyme (93 %).

**Table 1:** Comparison between expression systems for the encapsulation of CYPBM3 inside P22

Expression system (plasmids)	CYP/capsid^a^	Total CYP^b^ Abs_280_ (µM)	Active CYP CO assay^c^ (µM)	% active CYP^d^ (CO/Abs_280_)
Co-expression (pETDuet)	156.0 (±0.4)	123.5	9.2	7
Differential expression 1 (pBAD + pRSF)	129.5 (±0.1)	135.3	31.8	23
Differential expression 2 (pBAD + pRSF)	109.7 (±2.8)	123.7	42.9	35

^a^Determined by Eq. 1

^b^Total CYP determined using an extinction coefficient at 280 nm assuming a molar extinction coefficient ε_280_ = 44,920 M^−1^ cm^−1^ for coat protein and ε_280_ = 52,830 M^−1^ cm^−1^ for CYP-SP (theoretically calculated using ProtParam, Gasteiger, 2005). The concentration of protein was calculated using the Lambert–Beer equation, Abs_T_ = C_CP·_ɛ_CP_l + C_CYP-SP·_ɛ_CYP-SP_l as described previously [15]

^c^Active CYP determined by the formation of CO-CYP complex in reducing medium with a extinction coefficient of ε_450_ = 0.091 nM^−1^cm^−1^ [26]

^d^Active/total CYP ratio expressed in percentage

Differential expression was performed using two different strategies. In the first case, 0.2 % l-arabinose was used to induce expression from the CYP-SP gene and 0.5 mM IPTG to induce the CP gene expression. In the second case, 0.125 % l-arabinose was used to induce expression of the CYP-SP gene and 0.3 mM IPTG to induce the CP gene. By lowering the concentration of inducers we were able to increase the percentage of active CYP without significantly affecting the amount of encapsulated enzyme per capsid (Table 1). Thus, in the differential expression strategy the number of active enzymes per capsid could be improved by tuning expression parameters, such as induction time and concentration of inducers.

In order to determine if all CYP molecules were expressed as holoenzymes, and explain the significant difference in concentration values encountered by absorbance at 280 and the CO assay, the iron concentration present in encapsulated CYPs was determined by Inductively Coupled Plasma Mass Spectrometry (ICP-MS). Samples from the second differential expression strategy showed a complete incorporation of iron-heme cofactor (CYP:Fe molar proportion of 1:1) in all encapsulated CYPs, in which only 35 % were observed to be active by the carbon monoxide binding assay. This suggests that the other two-thirds of encapsulated enzyme could be trapped as misfolded intermediates where the iron of the heme group is bound, but in an incorrect coordination state [27]. This could be due to the fast interaction of CYP-SP with the CP, disrupting the CYP folding before the correct incorporation of the heme prosthetic group, as supported by the low proportion of active CYP found in the co-expression strategy (Table 1).

**Fig. 1:**
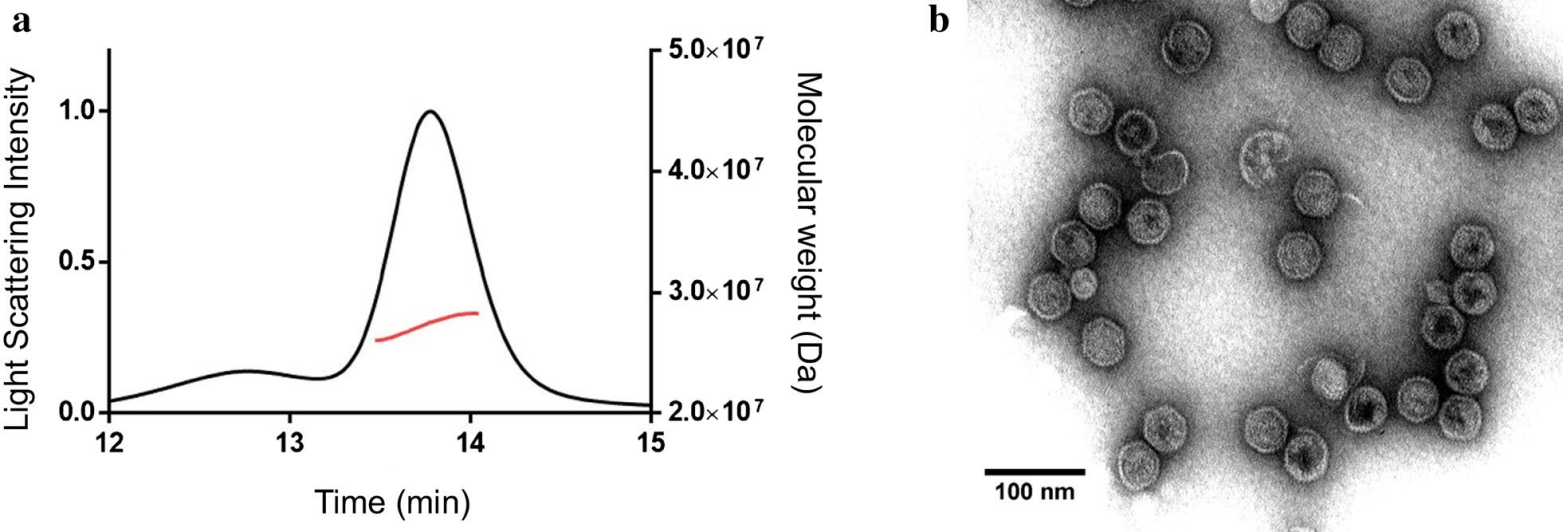
Structural characterization of P22-CYP VLPs. **a** Size exclusion chromatogram with molecular weight analysis by HPLC-MALS-RI. *Black line* light scattering intensity. *Red line* molecular weight (Da). **b** Transmission electron micrograph of P22 capsids with encapsulated CYP

The biocatalytic VLP preparations, with the highest proportion of active CYP, corresponding to the second strategy (differential expression) were further characterized structurally and kinetically. VLPs were produced in high yields of 120 mg of P22-CYP VLPs per liter of culture, and were easily purified. Highly monodisperse VLP preparations were obtained, with an average VLP diameter of 53.6 ± 0.2 nm as determined by HPLC-MALS-RI. The amount of encapsulated CYP per capsid was 109.7 ± 2.8 CYP molecules per particle as determined by HPLC-size exclusion chromatography coupled to in-line multi-angle laser light scattering (MALS) and refractive index (RI) detectors (Fig. 1a). As confirmed by TEM, the nanoparticles are quasi-spherical and well structured, suggesting that the packaging of the enzyme did not have any repercussions on the assembly of the P22 capsid (Fig. 1b).

Compared to other recently designed nanovehicles for enzyme intracellular delivery, P22-CYP VLPs showed a higher enzyme content of 40 % (w/w) of the total nanoparticle weight, demonstrating a higher payload capacity, compared to 16.7 % in a superoxide dismutase-mesoporous silica nanoparticle system [28] and 15 % in a nanoparticle formed by the conjugation of β-galactosidase with enhanced green fluorescent protein [29].

The catalytic constants for the encapsulated and free CYPBM3 were determined (Table 2). For both preparations, only the catalytically active enzyme was considered for the k_cat_ and K_M_ calculations. The catalytic constants are apparent since we could not reach the saturation concentration of peroxide. The enzyme was rapidly inactivated in the presence of high concentrations of hydrogen peroxide (>60 mM), as previously reported [21]. In addition, at such high peroxide concentrations, substrate oxidation seems to be affected by a possible Fenton-like reaction (data not shown).

**Table 2:** Apparent catalytic constants for free and encapsulated CYPBM3

	k_cat_^a^ (min^−1^)	K_M_ (H_2_O_2_) (mM)	k_cat_/K_M_ (min^−1^ mM^−1^)
P22-CYP	507.9 (±37.1)	25.2(±4.2)	20.1
Free CYP	720.5 (±27.8)	18.5 (±1.8)	38.9

^a^The reaction mixture contained a catalytic saturating concentration of 500 µM 2,6-DMP

Encapsulated CYP showed a 70 % catalytic rate (k_cat_) compared to that found with the free enzyme, while its affinity constant (K_M_) for hydrogen peroxide was slightly higher when compared to the free enzyme. Thus, the catalytic efficiency (k_cat_/K_M_) found for the encapsulated enzyme is around half of the free enzyme. The diffusion coefficients of P22-CYP VLPs and free CYP were calculated according to [30] using a hydrodynamic radius of 26.8 nm and 3.3 nm, respectively. The resulting diffusion coefficients were 8.06 × 10^−12^ m^2^ s^−1^ for P22-CYP and 6.55 × 10^−11^ m^2^ s^−1^ for the free enzyme.

The decrease in catalytic activity of encapsulated enzymes has been reported for other VLP-enzyme systems [13, 15, 17, 31], and has been mainly attributed to diffusional problems and to a decrease in the structural flexibility of enzymes as a result of a highly crowded space. The diffusion rate of substrates through VLP pores could affect their transformation rates. It depends on several factors such as the hydrodynamic radius of the substrates, the pore diameter and the electrostatic environment around the capsid pores, as well as the degree of obstruction of the pores by the cargo enzyme. Comellas-Aragones et al. [12] showed a diffusion time increase of almost three orders of magnitude for rhodamine when it had to pass through the 2 nm pores present in CCMV capsids, when compared to the substrate diffusion time in distilled water. Another limiting factor related to mass transfer phenomena is the decrease, by one order of magnitude, of the diffusion coefficient of P22-CYP compared to the free enzyme. This likely affects the rate of collisions between enzyme and substrate molecules influencing the kinetics.

On the other hand, the high degree of confinement found inside the capsids, in our case M_conf_ = 3.14 mM (capsid internal volume of 5.8 × 10^−20^ L), could restrict the conformational changes needed to perform catalysis. It is well known for this particular CYP that the F and G helices undergo important structural motion while executing catalysis [32] that could be impaired by the high degree of confinement. A decrease in activity using other immobilization materials, such as sol–gel matrices and DEAE resins, has also been reported for the heme domain of CYPBM3 as well for the whole protein [33, 34] suggesting that this enzyme is sensitive to motion impairment. The fact that CYPBM3 was expressed as a fusion protein with a fragment of the P22 scaffold protein could also have affected its kinetic behavior. Further evaluation of the structural dynamics of this CYP inside the P22 capsid are needed to better understand and explain the particular changes observed in the catalytic parameters. Moreover, given the importance that diffusion may impose in the system for relevant therapeutic treatments this issue should be assessed.

**Fig. 2:**
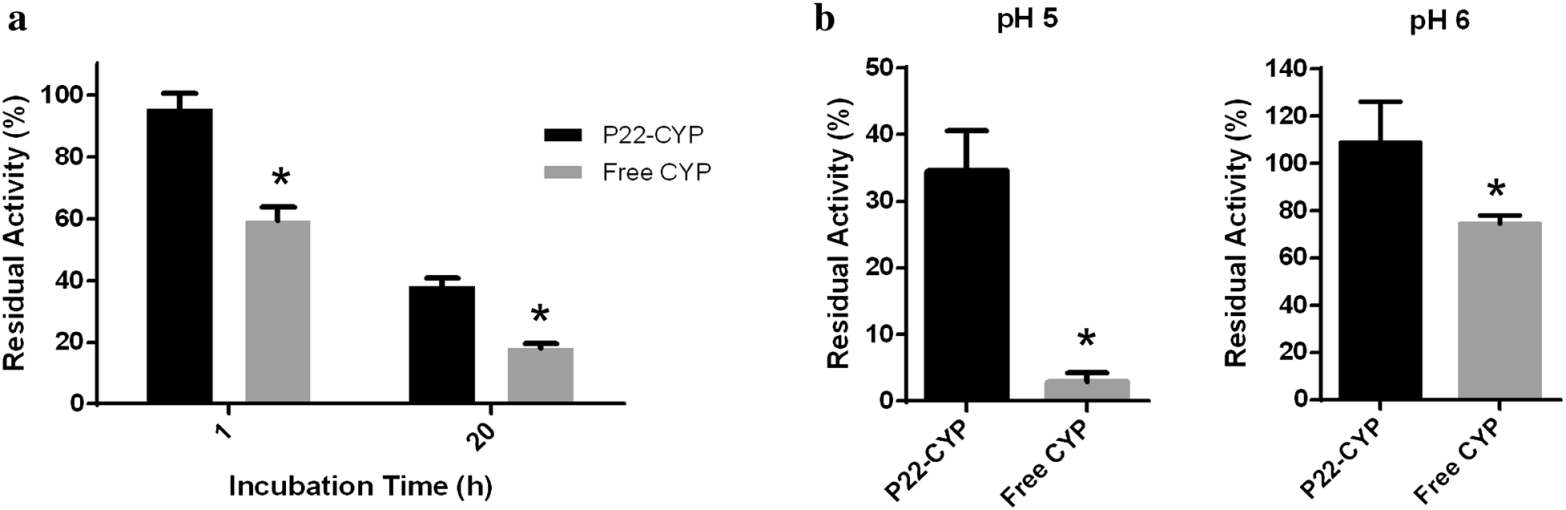
Comparison between free and encapsulated CYP against protection from protease inactivation and acidic pH stability. **a** Residual CYP activity after incubation of P22-CYP and free CYP with 10 U of trypsin per mg of protein during 1 and 20 h. **b** Residual CYP activity after incubation of P22-CYP and free CYP in pH 5 and pH 6 for 1 h. CYP activity was measured at 100 mM Tris–HCl pH 8 buffer using 2,6-DMP as a substrate and 5 mM of H_2_O_2_ to initiate the reaction. Treatments were analyzed by one-way ANOVA and multiple comparisons between mean values using Turkey’s test. All values showed to be statistical different (*p < 0.01)

Due to their confinement, it could be expected that enzyme stability against therapeutically important factors could increase. Enzyme stability towards protease degradation and to pH were assayed (Fig. 2). CYPBM3 encapsulation inside P22 capsid confers protection of the cargo enzyme against proteases. After 1-h incubation in the presence of trypsin, the residual activity of the encapsulated CYP was 90.3 % while the free enzyme retained only 59.5 % of its original activity. After 20 h of incubation with the protease, the retention of activity for the P22-CYP was 36.1 %, while the free enzyme retained only 18.2 % (Fig. 2a). This intrinsic capacity of viruses to protect their cargo from proteolytic degradation can be exploited in the use of these VLPs as enzyme delivery vehicles for therapeutic purposes, ensuring a higher lifetime of cargo in vivo. Moreover, this increase in the proteolytic stability is particularly important in cancer therapy. It is well known that there is an overexpression of extracellular matrix metalloproteinases (MMPs) in the tumor microenvironment. The MMPs are involved in cell growth, tissue invasion and metastasis, angiogenesis and migration, among others processes, and they role is to cleavage a variety of extracellular matrix components (adhesion molecules), growth-factor-binding proteins, growth-factor precursors, receptor tyrosine kinases, cell-adhesion molecules and other proteases [35]. The proteolytic stability of VLPs has been reported [36, 37] and could be originated by less accessible hydrolysis sites due to new bond formation in the compact VLP structure. It is important to point out that for further in vivo experiments, the VLPs will be covalently covered with PEG that, in addition to render them less immunogenic, it could be envisaged a higher proteolytic stability.

Another advantage of CYPBM3 confinement inside the P22 viral cage is an increase in stability towards acidic pH, where the free CYP precipitates. The encapsulation of the enzyme prevents its precipitation at pH 5 and retains, after 1-h incubation, 33.3 % of its original activity, while the free soluble CYP retains only 2.9 % of activity. After 1-h incubation at pH 6, the encapsulated CYP retains full activity, while the free preparation loses 25 % of its original activity (Fig. 2b). The isoelectric point of the free CYPBM3-His Tag was 5.4 (experimentally calculated; data not shown), while the CYP-SP had a pI = 6.1 (theoretically calculated using ProtParam), this shift in pI could help to prevent the precipitation of the encapsulated enzyme. Also, the encapsulation of the enzyme within the capsid avoids bulk precipitation and subsequent aggregation of the CYPBM3, therefore retaining a higher proportion of activity. This increased stability towards acidic pH is of particular importance from a therapeutic point of view, since the majority of nanoparticles are internalized by cells through an endocytic pathway, with early endosomes having a pH in the range of 6–6.5 and late lysosomes having a pH between 4.5 and 5.5 [38]. Moreover, the extracellular environments of tumors are known to be acidic (6.5–6.9) due to the increase in fermentative metabolism [39]. Thus, the effect of acidic pH on CYP can be relieved by its encapsulation inside the P22 capsid.

**Fig. 3:**
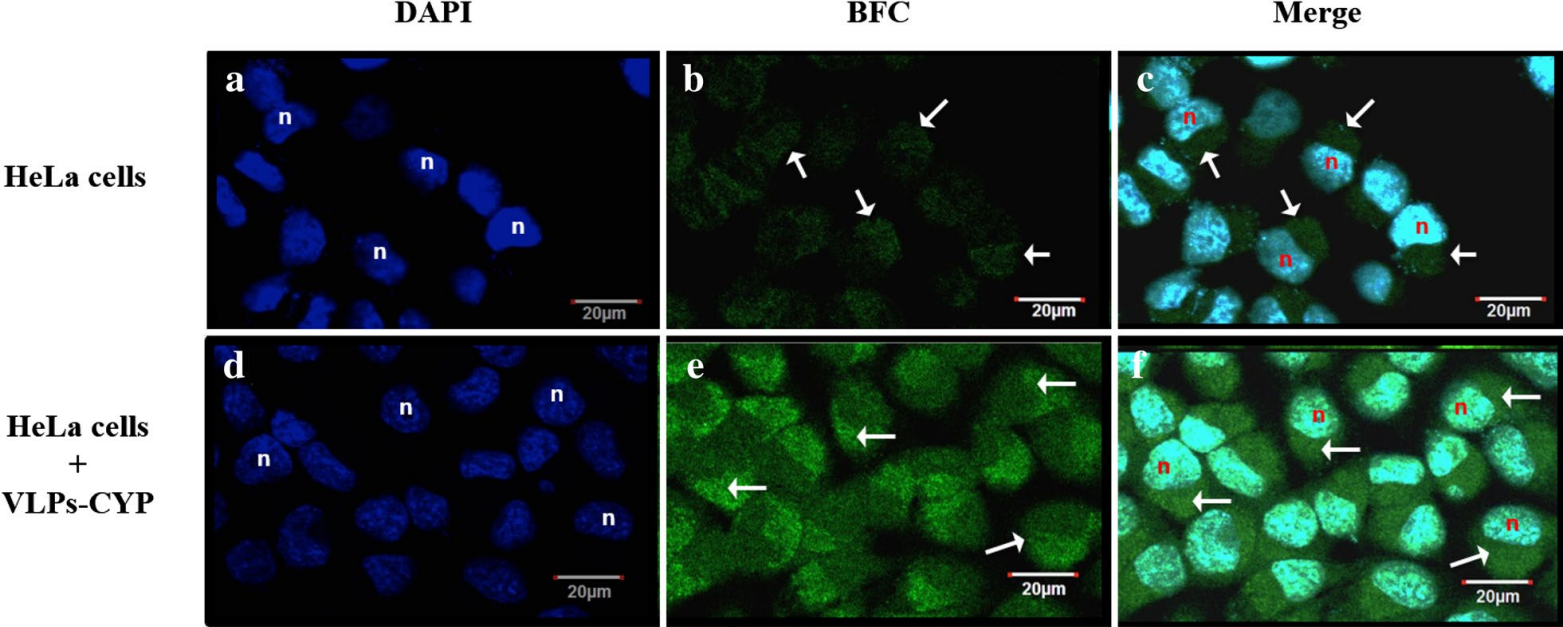
Cytochrome P450 activity assay in human cervix carcinoma cell line (HeLa). Staining with DAPI show nuclei of HeLa cells labeled as “n”, *panels* **a** and **d**. Endogenous CYP activity over BFC reagent was visualized in HeLa cells as observed in *panel* **b**. CYP activity of transfected VLPs-CYP nanoparticles in HeLa cells is shown in *panel* **e**. Overlay of DAPI and BFC localize the CYP activity in the cytoplasm of HeLa cells (*white arrows*), *panels* **c** and **f**. *Scale bar* represents 20 μm. Cells were visualized with a ×63 (DIC), 1.4 N.A. planapochromatic oil immersion objective

To evaluate whether P22-CYP VLPs were suitable vehicles for the intracellular delivery of cytochrome P450 activity, HeLa cells were transfected with the biocatalytic nanoparticles, which were then tested for enzymatic activity using 7-benzyloxy-4-trifluoromethylcoumarin (BFC) as a substrate. When BFC is metabolized into 7-hydroxy-4-trifluoromethylcoumarin (HFC) it produces an easily detectable fluorescent product (Fig. 3). The fluorescence intensity of cells treated with P22-CYP was significantly higher when compared to those observed from endogenous CYP activity in untreated HeLa cells. BFC processing into the fluorescent HFC reagent was clearly localized in the cytoplasmic region as expected, since lipofectamine was used for nanoparticle internalization given the lack of P22 surface functionalization for mammalian cell uptake.

**Fig. 4:**
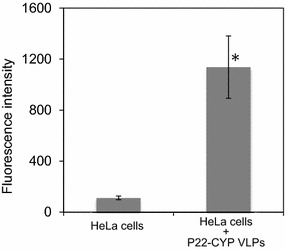
Cytchrome P450 enzymatic activity of transfected HeLa cells. The activity of endogenous CYP and lipofected P22-CYP VLP in HeLa cells was measured by the transformation of BFC reagent into the fluorescent HFC compound. Intensity in fluorescence was obtained in both cases from a 200,000 cells suspension with an excitation/emission spectra at 254/510 nm. Stastistical significance was analyzed by the Student’s t test (*p < 0.01)

In addition, CYP activity was quantitatively determined as fluorescence intensity. BFC is a specific substrate for CYP and it is transformed to 7-hydroxy-4-trifluoro-methylcoumarin (HFC) that produces an intense fluorescence at 510 nm. As expected, the control cells showed a basal endogenous CYP activity (110 ± 14 a.u.) while the P22-CYP transfected cells showed 10-times higher CYP activity (1136 ± 244 a.u.) (Fig. 4). Experiments with free CYP in the same reaction conditions showed a linear correlation between fluorescence of HFC and CYP activity. This result demonstrates the capacity of VLPs to deliver enzymatic activity to cells. With the increase of CYP activity in tumor cells, it is expected a better pro-drug activation in the target tissue and thus a more effective chemotherapy. This is important because the drug dose could be diminished, reducing the drastic side effects of treatment. Tamoxifen is metabolized to endoxifen, which has high affinity for estrogen receptor with the desired chemotherapeutic effect, but norendoxifen does not have the desired effect with extreme side effects. Approximately 20 % of the population has low CYP2D6 activity that renders tamoxifen less effective. The cellular uptake of tamoxifen by MCF-7 breast cancer cells is known [40] and because the estrogen receptors are located in the periphery of the nuclear membrane, these nanoparticles could provide increased therapeutic benefit by transforming tamoxifen to the active drug inside cells and binding to ER receptors.

Future studies on these biocatalytic P22 VLPs will be required to functionalize the outer surface of the capsid with specific ligands to target specific cells and trigger internalization as well as to bypass the immune system. Fortunately, there are abundant strategies being developed to target nanoparticles to specific cell linages [41–43] that are compatible with VLP functionalization.

## Conclusions

The P22 viral nanostructures are remarkable protein containers, as demonstrated in this study, where biocatalytic VLPs were generated based on the directed encapsulation of CYPBM3 “21B3”. This nanobioreactor contains a considerable amount of CYP per capsid. The CYP cargo retains 70 % of the catalytic activity, and showed a slightly higher K_M_ as compared to the free enzyme. New unique properties, including protease resistance and stability in acidic pH, were generated through the encapsulation of the CYP inside the P22 capsid. These two improved characteristics of the biocatalytic VLP over the free enzyme, along with a great payload capacity and the successful proof of concept of cytochrome P450 enzymatic delivery in mammalian cells, are attractive for the potential use of these nanoreactors as enzymatic delivery systems for future therapeutic applications.

This potent CYP nanobioreactor can be exploited for enzyme prodrug therapy, particularly to activate anticarcinogeneic drugs into its active metabolites since it outperforms the generally low activities of human CYP. The P22-CYP nanobioreactor also provides a unique model for the study of kinetics in highly constrained environments, such as the one found inside the VLP.

## Methods

### Materials

DNA primers and DNA sequencing were obtained from Eurofins MWG Operon (Huntsville, AL). *E. coli* BL21(DE3) and 10G electrocompetent *E. coli* cells were purchased from Lucigen (Middleton, WI). Pfu Ultra DNA polymerase was obtained from Agilent Technologies (Santa Clara, CA). T4 DNA ligase and restriction enzymes NcoI, BamHI and SacI were purchased from New England Biolabs (Ipswich, MA). MiniElute Reaction Cleanup Kit, QIAquick Gel Extraction Kit and QIAprep Spin Miniprep kit were acquired from Qiagen (Valencia, CA). Hydrogen peroxide, 2,6-dimethoxyphenol (2,6-DMP) and 7-benzyloxy-4-(trifluoromethyl)-coumarin (BFC) were purchased from Sigma-Aldrich (St. Louis, MO). Trypsin from bovine pancreas (Type I, ~10,000 BAEE units/mg protein) was obtained from Sigma-Aldrich (St. Louis, MO).

### Expression and purification of free CYPBM3 “21B3”

The plasmid pCWori encoding the heme domain of the CYPBM3 “21B3” was a kind gift from Prof. France Arnold from the California Institute of Technology (Caltech). The CYPBM3 mutant 21B3 was expressed in *E. coli* using the β-d-thiogalactopyranoside (IPTG)-inducible pCWori vector as previously described [21]. CYPBM3 “21B3” purification was performed by chromatography in an EconoSystem from Bio-Rad equipped with a 5 mL Ni-pre-charged HisTrap HP column (Amersham Biosciences). The equilibration buffer consisted in 50 mM NaH_2_PO_4_, 300 mM NaCl and 10 mM imidazole, pH 8. The protein mixture was loaded at 1.5 mL min^−1^. The CYP protein was eluted in a buffer containing 300 mM imidazole at 3 mL/min for 10 min. The colored fractions were collected, concentrated by ultrafiltration and stored at −20 °C in 50 mM Tris–HCl buffer, pH 8, containing 10 % glycerol. CYP protein concentration was determined by using the CO assay [26].

### Plasmid constructs

The CYPBM3 “21B3” gene was amplified by PCR from the plasmid pCWori CYBM3 using the forward primer, 5′-AAAAATCATGCCATGGCAATTAAAGAAATGCCT-3′ and reverse primer, 5′-AAAAAAGCGGGATCCAGTGCTAGGTGAAGGAA-3′.

The amplified gene product was digested with NcoI and BamHI (underlined in primer sequences, respectively) and ligated into the previously linearized pETDuet-1 assembler vector containing the truncated scaffold protein SP_141–303_ and the P22 coat protein [16]. The ligation reaction was transformed into 10G electrocompetent cells and colonies were screened by colony PCR and restriction enzyme digestion. Hits were sequenced (Huntsville, AL) to confirm the correct DNA sequence. Once the correct sequence was verified, the pETDuet CYP-SP+CP_P22_ plasmid (Amp^R^) was transformed into BL21(DE3) for the simultaneous expression strategy. The fusion CYPBM3 “21B3”—scaffold protein (CYP-SP) gene was subcloned from the pETDuet CYP-SP + P22 into the pBAD plasmid. The pETDuet CYP-SP + P22 plasmid and pBAD vector were both digested with NcoI and SacI. The digested products were ligated and transformed into 10G electrocompetent cells. Colonies were screened by colony PCR and restriction enzyme digestion. Hits were sequenced (Huntsville, AL) to corroborate for the right DNA sequence. Once the correct sequence was verified, the pETDuet CYP-SP + CP_P22_ plasmid (Amp^R^) and the pRSF P22 plasmid (Km^R^) were transformed into BL21(DE3) for the differential expression strategy.

### Simultaneous protein expression strategy

*E. coli* BL21(DE3) cells harboring the expression plasmid pETDuet CYP-SP+CP_P22_ were grown on Terrific Broth (TB) medium, supplemented with 0.5 mM thiamine and trace elements, at 37 °C and 180 rpm in the presence of ampicillin to maintain selection for the plasmid until reaching and OD_600_ = 0.8. At this point, 0.5 mM of isopropyl-β-d-1-thiogalactopyranoside (IPTG) and 1 mM δ-aminolevulinic acid were added. Cell cultures were grown for additional 5 h at 30 °C and 135 rpm, then cells were harvested by centrifugation and the pellets stored at −20 °C overnight until purification.

### Differential protein expression strategy

*E. coli* BL21(DE3) cells harboring the expression plasmids pBAD CYP-SP and pRSF P22 were grown on TB medium, supplemented with 0.5 mM thiamine and trace elements, at 35 °C and 150 rpm for 7 h in the presence of ampicillin and kanamycin to maintain selection for both plasmids. At this point, two different induction schemes were followed: (1) The CYP-SP gene was induced first with 0.2 % of l-arabinose and the culture was supplemented 1 mM δ-aminolevulinic acid. The cell culture was grown for 16 h. After this period of time, the CP_P22_ gene was induced with 0.5 mM of IPTG and the cultures were grown for 3 additional hours at 30 °C and 150 rpm, then cells were harvested by centrifugation and the pellets stored at −20 °C. (2) The differential expression was performed as stated above but the expression of the CYP-SP gene was induced with 0.125 % of l-arabinose and the P22 with 0.3 mM IPTG.

### P22-CYP VLP purification

Cell pellets were resuspended in lysis buffer (50 mM sodium phosphate, 100 mM sodium chloride, pH 7.6) and lysed by sonication. Cell debris were removed by centrifugation at 12,000×*g* for 45 min at 4 °C. P22 VLPs were purified from the supernatant by ultracentrifugation over a 35 % (w/v) sucrose cushion and spun at 215,041×*g* on a Sorvall WX Ultra 80 ultracentrifuge (Thermo Scientific) for 50 min at 4 °C. The resulting P22 VLP pellet was resuspended in PBS (50 mM sodium phosphate, 25 mM sodium chloride, pH 7.0) and then purified over a 60 × 1.6 cm HiPrep 16/60 Sephacryl S-500 size exclusion column (GE Helathcare) using an AKTA Pharmacia FLPC. Flow rate for SEC purification was 1 mL min^−1^ of PBS. Fractions taken from SEC containing P22 VLPs were concentrated by ultracentrifugation at 215,041*g* for 50 min at 4 °C and the resulting capsid containing pellet was resuspended in 100 mM Tris–HCl pH 8 buffer. The purity of VLPs has been verified by gel electrophoresis and transmission electron microscopy (TEM). P22 VLPs concentration was determined by UV absorption at 280 nm using a molar extinction coefficients of ε_280_ = 44,920 M^−1^ cm^−1^ for coat protein and ε_280_ = 52,830 M^−1^ cm^−1^ for CYP-SP (theoretically calculated using ProtParam, [44]). The total concentration of protein in the P22 capsid with encapsulated CYP-SP (P22-CYP VLPs) was calculated using the Lambert–Beer equation, Abs_T_ = C_CP_·ɛ_CP_·l + C_CYP-SP_·ɛ_CYP-SP_·l as described previously [15]. Abs_T_ is the total absorbance of the sample measured at 280 nm.

### Size exclusion chromatography with multiangle light scattering and refractive index detection (HPLC-MALS-RI)

Samples were separated through a WTC-100S5 (Wyatt Technologies) size exclusion column utilizing an Agilent 1200 HPLC. Elution was performed at flow rate of 0.7 mL min^−1^, for a total run time of 25 min using 50 mM phosphate pH 7.2 buffer containing 100 mM sodium chloride and 200 ppm sodium azide. Samples of 25 µL (1 mg mL^−1^ concentration) were loaded into the column and the total run time was 30 min. Samples were detected using a UV–Vis detector (Agilent), a Wyatt HELEOS Multi Angle Laser Light Scattering (MALS) detector, and an Optilab rEX differential refractometer (Wyatt Technology Corporation). The molecular weight of the P22-CYP VLPs, the polydispersity of the sample as well as the hydrodynamic radius of the particle was calculated with Astra 5.3.14 software (Wyatt Technology Corporation).

The number of enzymes encapsulated within a P22 VLP was calculated using the following formula

1 $$CYP - SP_{per capsid} = \frac{{M_{(P22 capsid + CYP - SP)} - M_{(capsid)} }}{{M_{(CYP - SP)} }}$$

where M_(P22capsid+CYP-SP)_ is the molecular weight of the capsid with the encapsulated CYP-SP (determined experimentally with HPLC-MALS-RI), M_capsid_ is 19,572 kDa (46.6 kDa × 420 subunits) and M_CYP-SP_ is 71.5 kDa (calculated with Serial Clones 2.6, Franck Perez, Serial Basics).

### Transmission electron microscopy

VLPs (10 µL, 0.1 mg mL^−1^) were applied to carbon-formvar coated grids and incubated for 1 min, excess sample was removed with Whatman filter paper. Grids were then washed with 10 µL of distilled water, removing away liquid shortly after addition with filter paper and stained with 5 µL 1 % uranyl acetate for 1 min. Excess stain was removed by blotting with filter paper. Samples were analyzed with a LEO 912AB transmission electron microscope operated at 100 kV.

### Inductively coupled plasma-mass spectrometry (ICP-MS)

A P22-CYP VLP sample (21.6 mg) was incubated in concentrated nitric acid (HNO_3_) for 16 h at 70 °C. After sample mineralization, it was diluted with water to a final HNO_3_ concentration of 5 % in a final volume of 50 mL. Samples were sent to Energy Laboratories, Inc (Billings, MT, USA) to be analyzed. Sulfur was used as internal reference (8401 sulfur atoms per P22-CYP capsid) for determining the ratio of iron to protein.

### Free CYP and P22-CYP kinetic assays

The CYP enzymatic activity was determined by the transformation of 2,6-dimetoxyphenol (2,6-DMP) and spectrometrically monitored at 468 nm (ɛ_468_ = 14,800 M^−1^ cm^−1^) using an Agilent 8453 UV-Vis spectrophotometer. All reactions were performed in 50 mM Tris–HCl buffer (pH 8) at room temperature in a final volume of 0.1 mL. For the determination of catalytic parameters the concentration of 2,6-DMP was fixed at 500 µM. The reaction was initiated by adding H_2_O_2_ in a range between 1 and 60 mM. Catalytic constant values were obtained by fitting the data to a Michaelis–Menten equation (GraphPad Prism 6, GraphPad Software, Inc.).

### P22-CYP VLPs stability against acidic pH

The residual activity of free CYP and P22-CYP was measured at pH 5 (100 mM sodium acetate buffer) and pH 6 (100 mM potassium phosphate buffer), incubating the samples for 1 h in each buffer. Before determining enzymatic activity, samples were centrifuged for 3 min at 16,000*g*. The residual activity was measured in 50 mM Tris–HCl buffer (pH 8) at room temperature using 500 µM 2,6-DMP as a substrate and 5 mM H_2_O_2_ to initiate the reaction.

### P22-CYP VLPs protection against protease degradation

The encapsulated and free CYP were treated with 10 U of trypsin per 1 mg of enzyme and incubated for 1 and 20 h at room temperature in 50 mM Tris–HCl buffer (pH 8). After incubation, the residual activity was determined as stated above.

### Cell line and cell culture

Human cervix carcinoma cells (HeLa cells) were cultured in Dulbecco’s Modified Eagle’s Medium (DMEM) supplemented with 10 % Fetal Bovine Serum (FBS, BenchMark, Gemini Bio Products), 1 % Penicillin streptomycin (Sigma-Aldrich), 1 % l-glutamine and 1.5 g/l sodium bicarbonate. Cells were propagated in growth medium and maintained at 37 °C and 5 % CO_2_.

### P22-CYP VLPs transfection

Cell culture Petri dishes coated with Poly-d-lysine (MatTek P35GC1.5-10C) were used to seed 250,000 HeLa cells in DMEM media and incubated overnight at 37 °C and 5 % CO_2_. Transfection of P22-CYP nanoparticles was achieved using Lipofectamine 2000 reagent (Life technologies), according to [45] with few modifications to the manufacture’s protocol. Briefly, 3 µl of Lipofectamine 2000 was diluted in 100 µl of DMEM media without antibiotic and FBS (DMEM-SF) and 3.14 × 10^11^ P22-CYP nanoparticles were mixed with 100 µl of DMEM-SF media, both preparations were pre-incubated for 15 min at room temperature (RT). Afterwards, both samples were mixed for 30 min at RT. Prior to the addition of this transfection mixture to HeLa cell culture; cells were rinsed twice with sterile PBS buffer. The transfection mixture was added slowly on the top of the cell culture and let stand for 30 min at RT, then 1.8 ml of DMEM-SF media was added into HeLa cells culture and incubated for 4 h at 37 °C and 5 % CO_2_. After incubation, cell media was removed and the culture was rinsed once with PBS and 2 ml of complete DMEM media was added. HeLa cells transfected with VLP-CYP nanoparticles were incubated overnight at 37 °C and 5 % CO_2_.

### P22-CYP VLP enzyme activity in vitro assay

CYP enzyme activity was assayed in HeLa cells (endogenous CYP activity) and in HeLa cells transfected with P22-CYP VLPs. The CYP activity was estimated by the transformation of 7-benzyloxy-4-trifluoromethylcoumarin (BFC) in the fluorescent product 7-hydroxy-4-[trifluoromethyl]-coumarin (HFC) according to [46] with some modifications. Briefly, cell culture media was discarded and 15 µl of 20 mM BFC diluted in 150 µl of complete DMEM media was added to each culture plate and incubated in darkness for 10 min at RT. Complete DMEM media was added up to 1.5 ml to each plate and further incubated for 30 min at 37 °C and 5 % CO_2_. Then, 4.5 µl of 1 mM of hydrogen peroxide was added to each culture and incubated for 10 min at 37 °C and 5 % CO_2_. Cell culture plates were rinsed three times with PBS before the addition of 2 mL of complete DMEM media was added to each plate and incubated for 2 h at 37 °C and 5 % CO_2_ for further imaging analysis.

### Confocal microscopy cell imaging

HeLa cell cultures treated with BFC reagent were fixed with 4 % formaldehyde-PBS solution at 4 °C for 15 min. After fixation, cells were permeabilized with 0.5 % Triton X/PBS for 15 min at 4 °C. Nuclear staining was achieved by incubated the cells with DAPI at 0.5 ng/ml in darkness for 10 min at RT, followed by five washes with PBS. Nuclear staining with DAPI was also visualized with an inverted laser-scanning microscope Olympus FluoView FV1000 (Japan) using an argon ion laser for excitation at 405 nm wavelength and filters for emission of DAPI. BFC transformation into the fluorescent reagent HFC was detected using the GFP filter channel (excitation at 488 nm and emission at 515–530 nm). Cells were visualized with a 63 × (DIC), 1.4 N.A. planapochromatic oil immersion objective. The imaging parameters used produced no detectable background signal from any source other than from BFC and DAPI. Confocal images were captured using MetaMorph software for Olympus.

### CYP activity quantification in HeLa cells transfected with P22-CYP VLPs

The enzymatic activity of CYP in non-transfected and transfected HeLa cells with P22-CYP VLPs was measured spectrofluorimetrically. The fluorescence intensity originated by CYP-catalyzed transformation of BFC into HFC was monitored. After transfection, media from P22-CYP transfected cells and non-transfected HeLa cells (control cells) was replaced by 1.5 ml of complete DMEM media containing 15 µl of 20 mM BFC and incubated under darkness for 30 min at 37 °C and 5 % CO_2_. Then, cell culture plates were rinsed three times with PBS before the addition of 4.5 µl of 1 M of hydrogen peroxide and incubated for 10 min at 37 °C and 5 % CO_2_. After incubation, cells were rinsed with PBS and harvested with trypsin/EDTA treatment. Pelleted cells were counted and diluted in PBS to obtain 200,000 cells. Fluorescence intensity from transfected and non-transfected HeLa cells was measured in a fluorescence spectrophotomerer (Hitahchi F-7000), using an excitation source at 254 nm and emission measurement at 510 nm.

## Abbreviations

BFC: 7-benzyloxy-4-trifluoromethylcoumarin
CCMV: cowpea chlorotic mottle virus
CP: virus coat protein
CYP: cytochrome P450
CYP-SP: fusion protein cytochrome P450-scaffold protein
HFC: 7-hydroxy-4-trifluoromethylcoumarin
HPLC-MALS-RI: high-performance liquid chromatography equipped with multi-angle laser light scattering (MALS) and refractive index (RI) detectors
IPTG: isopropyl-β-d-1-thiogalactopyranoside
P22-CYP: capsid nanoparticle containing cytochrome P450
SP: virus scaffold protein
VLP: virus-like particle
